# Transgenic mouse model for imaging of interleukin-1β-related inflammation *in vivo*

**DOI:** 10.1038/srep17205

**Published:** 2015-11-24

**Authors:** Takao Iwawaki, Ryoko Akai, Daisuke Oikawa, Takae Toyoshima, Mayuko Yoshino, Mitsumi Suzuki, Naoki Takeda, Tomo-o Ishikawa, Yosky Kataoka, Ken-ichi Yamamura

**Affiliations:** 1 Iwawaki laboratory, Education and Research Support Center, Graduate School of Medicine, Gunma University, 3-39-22 Showa-machi, Maebashi, Gunma 371-8511, Japan; 2 Laboratory of Molecular Cell Biology, Institute for Molecular and Cellular Regulation, Gunma University, 3-39-15 Showa-machi, Maebashi, Gunma 371-8512, Japan; 3 Division of Developmental Genetics, Institute of Resource Development and Analysis, Kumamoto University, 2-2-1 Honjo, Kumamoto 860-0811, Japan; 4 TransGenic Inc, 7-1-14 Minatojima-minamimachi, Chuo-ku, Kobe, Hyogo 650-0047, Japan; 5 Cellular Function Imaging Team, Division of Bio-function Dynamics Imaging, RIKEN Center for Life Science Technologies, 6-7-3 Minatojima-minamimachi, Chuo-ku, Kobe, Hyogo 650-0047, Japan; 6 Yamamura Project Laboratory, Institute of Resource Development and Analysis, Kumamoto University, 2-2-1 Honjo, Chuoku, Kumamoto 860-0811, Japan

## Abstract

Inflammation is a biological response associated with symptoms of various diseases, and its study is important in gaining an understanding of the pathological conditions of such diseases and in making strategic plans for promoting healing. It is therefore essential to develop technologies for the detection of inflammatory conditions. Interleukin-1β (IL-1β) is a proinflammatory cytokine produced and secreted mainly by monocytes and macrophages in response to inflammatory stimulation. The activation of IL-1β is regulated through transcriptional induction by the promoter and post-translational processing by the inflammasome. Here we have developed a reporter gene to monitor the activation status of IL-1β by using a dual regulation system and, by using the reporter gene, we have established a mouse model that permits low-invasive visualization of the inflammatory status. Previous reporter systems dependent on the transcription or processing of IL-1β show problems in terms of background noise or signal specificity. Our reporter system overcomes these weaknesses by combining advantages from regulation by a promoter and processing of IL-1β. Our mouse model detected specific physiological inflammation in the liver and pancreas caused by hepatitis or pancreatitis models, respectively. Our reporter gene and mouse model are therefore expected to become useful bioresources for future medical science.

Inflammation is an innate immune response that has been known since ancient times. It is characterized by five cardinal signs: pain, redness, swelling, heat, and immobility^1^. Although we can observe the signs of inflammation with the naked eye in some cases, a variety of imaging technologies have recently been used to comprehend the inflammatory response at the molecular and cellular levels. For example, infiltration of immune cells into adipose tissue in obesity has been analyzed by means of two-photon excitation fluorescence microscopy^2^, and the local dynamics of lipid inflammatory mediators *in vivo* have been visualized by imaging mass microscopy^3^. Some fluorescent and luminescent probes have also been invented to detect the *in vivo* activity of inflammation-responsive enzymes, such as MPO or COX2^4^,^5^. As inflammatory responses are finely regulated by multiple signal transduction and catalytic functions of various molecules, imaging techniques should be developed in a multidirectional manner for research on biological phenomena associated with inflammation.

Interleukin-1β (IL-1β) is a known proinflammatory cytokine, like tumor necrosis factor-α (TNF-α) or interleukin-6 (IL-6)^6^, that is produced and secreted by various cell types (mainly monocytes and macrophages) in response to inflammatory stimulation^7^. IL-1β is therefore regarded as a molecular marker of inflammation. The molecular mechanism for activation of IL-1β has been well studied. IL-1β has been shown to be regulated by the following two-step mechanism. In response to an inflammatory stimulation, gene expression of IL-1β is activated by transcription factors such as nuclear factor-κB (NF-κB) and activator protein-1 (AP-1)^8^. This transcriptional induction activates the production of a proIL-1β protein. Secondly, the proIL-1β protein is processed by caspase-1, activated in the inflammasome, and is converted into mature IL-1β protein that can be secreted into extracellular space^9^.

Many researchers detect and analyze IL-1β by ELISA methods or by quantitative PCR analysis, using protein or RNA collected from blood and tissues, respectively. These methods excel in terms of their quantitative capability, but they are unsuitable for the examination of real-time changes and local differences. On the other hand, *in vivo* imaging techniques should enable us to detect and measure the activity of IL-1β in a small region of tissue *in vivo* in real time. In fact, several research groups have previously reported that it is possible by using transgenic mice that express an IL-1β promoter-controled reporter gene^10^,^11^. Another recently developed imaging technique monitors the processing of proIL-1β protein by caspase-1 at the cellular and mouse level^12^,^13^,^14^. However, these methods have problem associated with background noise or signal specificity. To overcome these problems, we have developed a novel IL-1β reporter that combines advantages from transcriptional regulation and post-translational processing and we have established a transgenic mouse model by introducing the reporter gene to permit *in vivo* imaging of the inflammatory status.

## Results

### Design and construction of the IL-1β-based dual-operating luciferase gene

**Figure 1:**
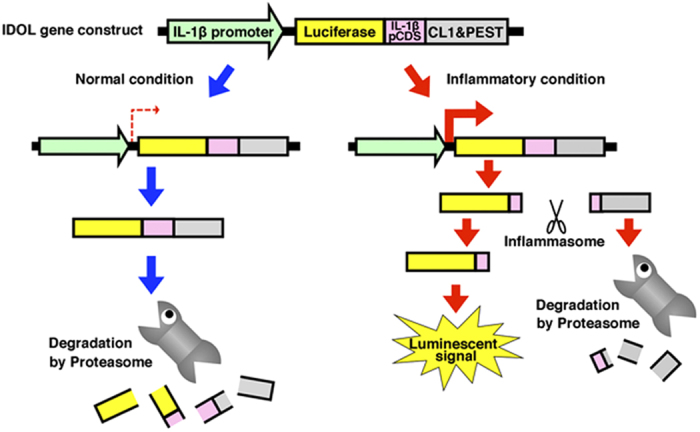
Schematic of the IDOL function. pIL1b (−5 k)-TKpro-GL4-HindIII-IL1b(17–216)-F-CLτEST were generated as a IDOL construct. A mouse IL-1β partial cDNA sequence (pink) links a luciferase cDNA sequence (yellow) to a CL1–PEST degradation signal (gray) in frame. This fusion gene is transcriptionally controlled by the mouse IL-1β promoter (green) in the IDOL construct. Transcription of the fusion gene should not, therefore, be induced under normal conditions. Even if the transcript of the fusion gene is partially translated into the fusion protein with the CL1–PEST degradation signal, it should be degraded by the proteasome. On the other hand, transcription of the fusion gene should be robustly induced under inflammatory conditions. The protein translated from the transcript of the fusion gene should be processed by the inflammasome, and be divided into a lucifrease reporter region and a CL1–PEST degradation signal region. The luciferase protein region should be stabilized by release from the CL1–PEST degradation signal, and should emit an intense luminescent signals. To facilitate Western blot analysis of the new reporter, a Flag tag sequence is also inserted at the join between the mouse IL-1β partial coding region and the CL1–PEST degradation signal in the IDOL gene.

To detect the activation of IL-1β faithfully, we designed a reporter gene that is subject to dual regulation by transcription of the gene promoter and by processing through the inflammasome (Fig. 1). A luciferase gene was fused with a mouse IL-1β partial coding region, which includes the inflammasome-dependent processing site (D^117^/V^118^), and with the CL1–PEST degradation signal. CL1 and PEST have been previously reported as a degradation signal recognized by the ubiquitin-proteasome system^15^,^16^. The fusion gene was then ligated downstream of the mouse IL-1β promoter to permit regulation at the transcriptional level. Under normal conditions, the fusion gene should not be transcriptionally induced and the leaked fusion protein should be degraded by the CL1–PEST degradation signal; consequently, little signal should be detected. However, under inflammatory conditions, the fusion gene should be induced at the transcriptional level by endogenous transcription factors such as NF-κB and AP-1. The resulting protein should then be processed by the inflammasome, and be divided into a lucifrease reporter region and a CL1–PEST degradation signal region. The luciferase protein region should be stabilized by separation from the CL1-PEST degradation signal, and should emit an intense signal (Fig. 1).

**Figure 2:**
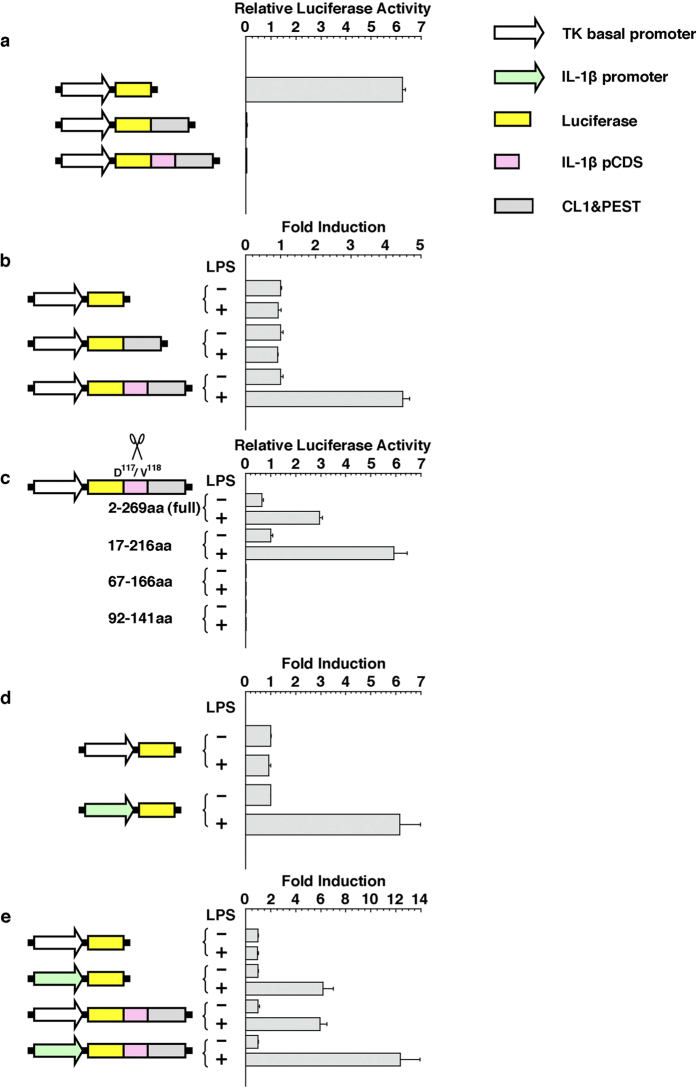
Reporter assay to confirm the functionality of each component of the IDOL gene. Reporter constructs containing all the relevant components were transfected into RAW264 cells. The transfectants were treated in the presence or absence of LPS for 6 h before the reporter assay. (**a,b**) Confirmation of the functionality of the mouse IL-1β partial cDNA sequence and the CL1–PEST degradation signal under normal conditions (**a**) and under inflammatory conditions (**b**). (**c**) Optimization of the region for adoption as the mouse IL-1β partial cDNA sequence. (**d**) Confirmation of the responsiveness of mouse IL-1β promoter to inflammatory stimulation. (**e**) Confirmation of the higher responsiveness of the dual-regulated reporter to inflammatory stimulation. The results are shown as mean (column) ± S.E.M (error bar) from triplicate experiments in each reporter assay (**a–e**). Each value in (**a,c**) is shown as a relative luciferase activity normalized against internal standard. On the other hand, each value in (**b**,**d,e**) is shown as a fold induction normalized against either the sample without LPS.

Before constructing the reporter gene described above, we tested the functionality of each component of the reporter gene by using a mouse macrophage-like cell line, RAW264. This cell line is equipped for transcriptional activation and inflammasome-dependent processing of IL-1β in response to inflammatory stimulation^17^,^18^,^19^. First, we examined the effect of fusion of mouse IL-1β (2–269 amino acids (aa)) and the CL1–PEST degradation signal. Under noninflammatory conditions, the reporter activity of cells expressing the luciferase gene fused to the mouse IL-1β partial coding region and the CL1-PEST degradation signal (pTKpro-GL4-IL1b(2-269)-F-CL1PEST) was less than 1/100 of that of cells expressing the luciferase gene not fused to the mouse IL-1β partial coding region and the CL1–PEST degradation signal (pTKpro-GL4), and was comparable with that of cells expressing luciferase gene fused directly to the CL1–PEST degradation signal (pTKpro-GL4-F-CL1PEST) (Fig. 2a). On the other hand, the luciferase activity of cells transduced with pTKpro-GL4-IL1b(2-269)-F-CL1PEST was 4.5 times higher under inflammatory conditions than under noninflammatory conditions (Fig. 2b). Thus, the fusion with IL-1β and the CL1–PEST degradation signal suppressed background noise and imparted an inflammatory responsiveness to the reporter construct. Next, we examined the effect of the length of the IL-1β fused to the reporter. Among a series of reporter constructs containing the inflammasome-processing site of IL-1β (D^117^/V^118^), the fragment corresponding to 17–216 aa showed a somewhat better responsiveness than the full-length (2–269 aa) IL-1β, whereas fusions with 67–166 aa or 92–141 aa fragments showed no luciferase activities (Fig. 2c). On the basis of these results, we used the region corresponding to 17–216aa as a IL-1β partial fragment in the our subsequent experiments. Finally, we evaluated the promoter activity was evaluated. The reporter activity of cells expressing the luciferase gene controlled by the mouse IL-1β promoter (pIL1b(−5k)-TKpro-GL4) was 6 times higher under inflammatory conditions than under noninflammatory conditions, although the reporter activity of cells expressing the luciferase gene controlled the by herpes simplex virus type 1 thymidine kinase promoter (pTKpro-GL4) remained constant irrespective of the presence or absence of inflammatory stimulation (Fig. 2d). Thus, transcriptional control by the promoter of IL-1β gene also, as expected, provided inflammatory responsiveness to the reporter construct.

Having confirmed the functionality of each component of our new reporter gene from the preliminary experiments described above, we assembled each of the devices to construct the new reporter gene, and we compared it with some prototype reporters in terms of its responsiveness to inflammatory stimulation. The luciferase activity of cells transduced with the new reporter gene (pIL1b (-5k)-TKpro-GL4-HindIII-IL1b(17–216)-F-CL1PEST) was 12 times higher under inflammatory conditions than under noninflammatory conditions, whereas the reporter activity of cells transduced with prototypes (pTKpro-GL4-IL1b(17–216)-F-CL1PEST and pIL1b(−5k)-TKpro-GL4) was only 6 times higher under inflammatory conditions than under noninflammatory conditions (Fig. 2e). These results therefore indicate that our new reporter gene is superior to the prototypes in terms of its responsiveness to inflammatory stimulation. We have named the new reporter “IDOL” from IL-1β based Dual Operating Luciferase.

### Generation and characterization of IDOL transgenic mice

**Figure 3:**
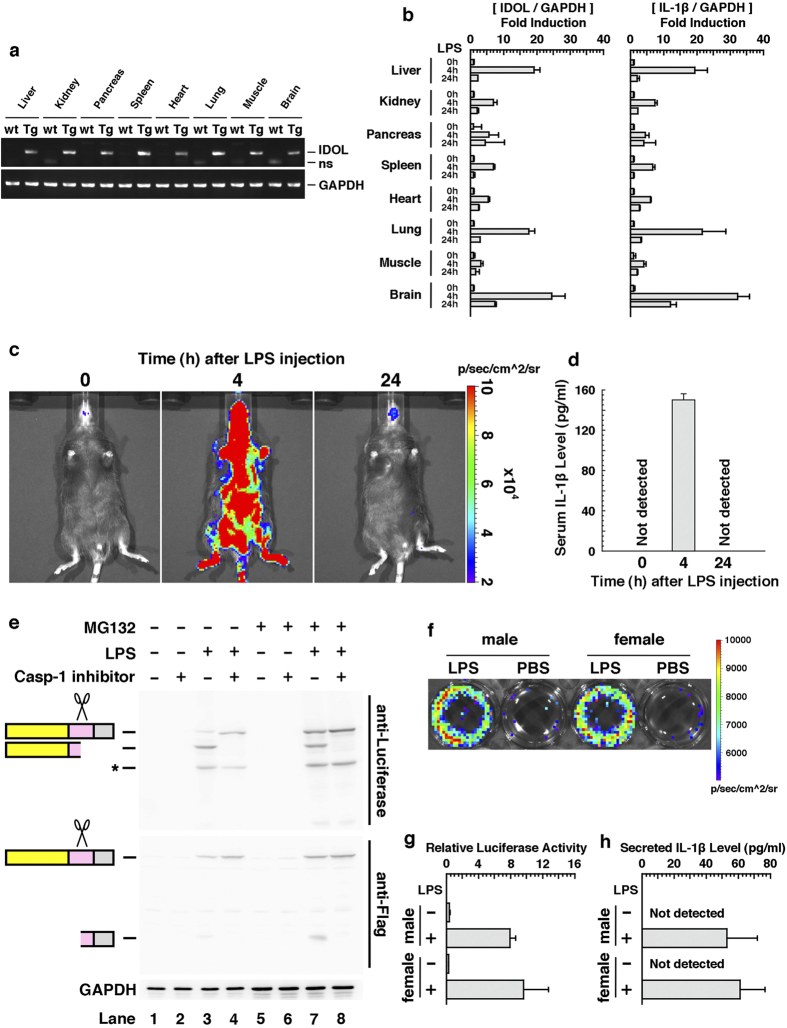
Characterization of IDOL transgenic mice. (**a**) RT-PCR analysis of IDOL transgene expression in various tissues. GAPDH was used as an internal standard. Nonspecific signals, ns; wild type, wt; transgenic, Tg. (**b**) Quantitative PCR analysis of the transgene and endogenous IL-1β in various tissues derived from IDOL mice treated with LPS for the indicated time. GAPDH was used as an internal standard. (**c**) Luminescent signals from IDOL mice treated with LPS for indicated time, obtained by *in vivo* imaging analysis at the whole-body level. The mice shown were identical. (**d**) Serum IL-1β level of IDOL mice identical to those examined in the imaging analysis (**c**). (**e**) Western blot analysis of CD11b^+^ splenic macrophage cells isolated from IDOL mice. CD11b^+^ cells were treated with the indicated drug before lysis for Western blot analysis. Nonspecific signals are shown as asterisks. GAPDH was used as a loading control. (**f,g**) Luminescent signals of CD11b^+^ cells isolated from IDOL mice, obtained by *in vitro* imaging analysis (**f**) or by a luciferase reporter assay (**g**). (**h**) Secreted IL-1β levels of CD11b^+^ cells isolated from IDOL mice. CD11b^+^ cells were optionally treated with LPS *in vitro*. The results in (**b**,**d**,**g,h**) are shown as mean (column) ± S.E.M (error bar) from triplicate experiments.

By using the IDOL gene, we generated IDOL transgenic mice. RT-PCR analysis revealed that the transgene was expressed in all the examined organs, even under normal conditions (Fig. 3a). Analysis by quantitative PCR also revealed that expression level of the transgene was induced 4 to 25 times in each organ under inflammatory conditions compared with noninflammatory conditions, and that the expression pattern of the transgene was approximately coincident with that of endogenous IL-1β (Fig. 3b). By *in vivo* imaging analysis, systemic intense luminescent signals were detected from IDOL mice after inflammatory stimulation, whereas weak or no signals were detected before stimulation (Fig. 3c). The serum IL-1β levels of IDOL mice identical to those examined in the imaging analysis are shown in Fig. 3d. Comparison of these data showed that time course of the luminescent signals was also approximately coincident with that of endogenous IL-1β. As described in the Introduction, IL-1β is mainly produced and secreted by monocytes and macrophages in response to inflammatory stimulation. To confirm the functionality of the IDOL gene in those cells, we isolated CD11b^+^ splenic macrophage cells and we subjected them to Western blot analysis with an anti-luciferase antibody and an anti-Flag antibody. The assays revealed that the unprocessed form of the reporter protein was present during inflammatory stimulation, but hardly any was present during noninflammatory stimulation (Fig. 3e, lanes 1 and 3). The processed form was also detected under inflammatory conditions, but the signal was eliminated on treatment with caspase-1 inhibitor (Fig. 3e, lanes 3 and 4). Traces of the unprocessed form and the processed C-terminal fragment were detected during inflammatory stimulation, and these signals became intense on treatment with a proteasome inhibitor, whereas treatment with the proteasome inhibitor did not affect the signals from the processed N-terminal fragment (Fig. 3e, lanes 3 and 7). These results show that all the designed devices (promoter, processing site, and degradation signal) in IDOL play their expected roles in CD11b^+^ cells derived from IDOL mice. In fact, the measurement of luciferase activity revealed that the luminescent signals of CD11b^+^ cells was increased by 20 to 40 times during inflammatory stimulation compared with noninflammatory stimulation (Fig. 3f,g). The IL-1β levels in condition medium of CD11b^+^ cells identical to those examined in the imaging analysis are measured by ELISA and shown in Fig. 3h. These data indicate that IDOL signals reflect secretion of endogenous IL-1β in macrophage cells.

### Luminescent signals from IDOL transgenic mice with experimental hepatitis

**Figure 4:**
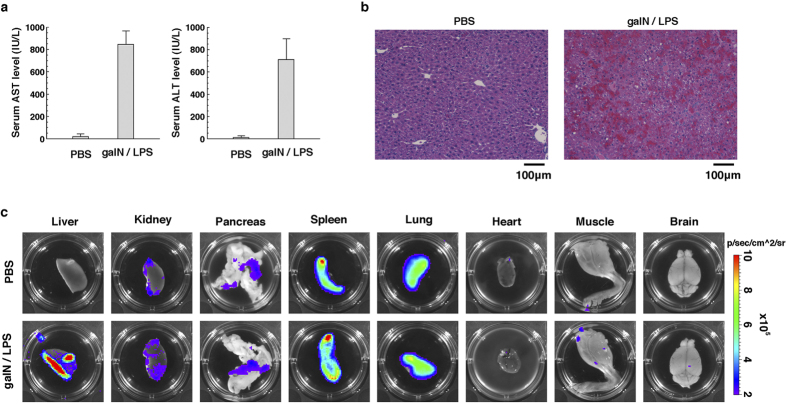
Luminescent signals from IDOL transgenic mice with experimental hepatitis. (**a**) Serum AST and ALT levels of IDOL mice injected with d-galactosamine/LPS or with PBS. Serum for measurement of AST and ALT levels was collected 6 h after the injection. The results are shown as mean (column) ± S.E.M (error bar) from triplicate experiments in each assay. (**b**) HE staining of liver tissue of IDOL mice injected with d-galactosamine/LPS or PBS. The liver tissues for HE staining were collected 6 h after the injection. (**c**) Luminescent signals of various tissues collected from IDOL mice by *ex vitro* imaging analysis. Each organ for *ex vitro* imaging analysis was collected 6 h after the injection.

To assess resource availability of IDOL mice in disease models, we examined the luminescent signals of IDOL mice in which hepatitis was induced by administration of d-galactosamine (2-amino-2-deoxy-d-galactopyranose). This amino sugar is known to induce acute hepatitis in mice on intraperitoneal injection with lipopolysaccharide (LPS)^20^. Levels of serum aspartate transaminase (AST) and alanine aminotransferase (ALT), which are typical indicators of hepatic damage, were actually increased by 40 to 60 times in IDOL mice injected intraperitoneally with a mixture of d-galactosamine and LPS compared with those injected with phosphate-buffered saline (PBS) as a negative control (Fig. 4a). The liver tissues derived from mice injected with d-galactosamine/LPS showed hemorrhage, infiltration of leukocytes, and hepatocyte death (Fig. 4b). These data indicate that, as expected, intraperitoneal injection of d-galactosamine/LPS induced acute hepatitis, even in IDOL mice. By *ex vivo* imaging analysis, intense luminescent signals were, as expected, detected in liver tissues from IDOL mice injected with d-galactosamine/LPS compared with those injected with PBS (Fig. 4c). In contrast, luminescent signals of other tissues were barely changed on injection with d-galactosamine/LPS, although basal luminescent signals were widely different among the various tissues (Fig. 4c).

### Luminescent signals from IDOL transgenic mice with experimental pancreatitis

**Figure 5:**
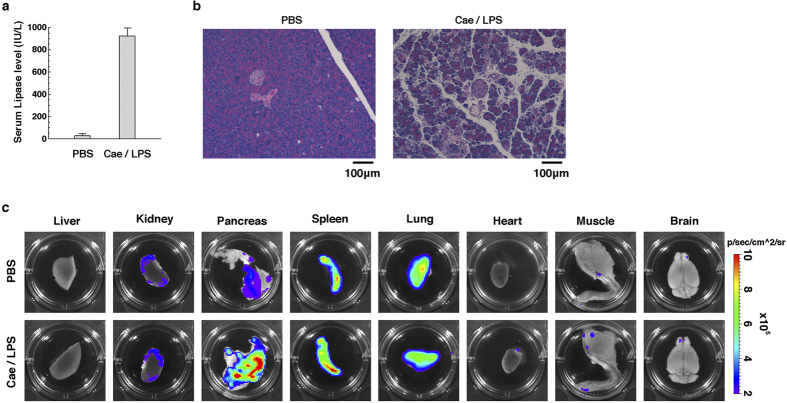
Luminescent signals from IDOL transgenic mice with experimental pancreatitis. (**a**) Serum lipase levels of IDOL mice injected with caerulein/LPS or PBS. Serum for measurement of the lipase level was collected 6 h after the first injection. The results are shown as mean (column) ± S.E.M (error bar) from triplicate experiments. (**b**) HE staining of the pancreatic tissues of IDOL mice injected with caerulein/LPS or PBS. The pancreatic tissues for HE staining were collected 6 h after the first injection. (**c**) Luminescent signals of various tissues collected from IDOL mice by *ex vitro* imaging analysis. Each organ for *ex vitro* imaging analysis was collected 6 h after the first injection.

Next, we examined the luminescent signals from IDOL mice in which pancreatitis was induced by administration of caerulein. This oligopeptide is known to induce acute pancreatitis in mice when injected intraperitoneally along with LPS^21^. The level of serum lipase, a typical indicator of pancreatic damage, was increased 30 times in IDOL mice injected intraperitoneally with a mixture of caerulein and LPS, compared with those injected with PBS as a negative control (Fig. 5a). The pancreatic tissues derived from mice injected with caerulein/LPS showed edema, infiltration of leukocytes, and acinar cell death (Fig. 5b). These results show that, as expected, intraperitoneal injection of caerulein/LPS induces acute pancreatitis, even in IDOL mice. By *ex vivo* imaging analysis, more-intense luminescent signals were, as expected, detected in the pancreatic tissues derived from IDOL mice injected with caerulein/LPS compared with those injected with PBS as a negative control (Fig. 5c). In contrast, luminescent signals from other tissues were identical in IDOL mice injected with caerulein/LPS and those injected with PBS (Fig. 5c). On the basis of these results of our experiments using hepatitis and pancreatitis models, the use of IDOL mice not only permits the detect of systemic inflammation but also permits the detection of tissue-specific inflammation elicited by disease models.

## Discussion

We have designed a new reporter system that converts activation of IL-1β into expression of luciferase (Fig. 1). This reporter system showed high responsivity to LPS in mouse macrophage-like cell lines (Fig. 2). In addition, transgenic mice transduced with the reporter gene showed an acute response to inflammatory stimulation, and displayed an induced transcriptional activity and signal activity of the reporter. The responsiveness of the reporter was approximately coincident with that of endogenous IL-1β. Furthermore, the regulation of processing and the degradation of the reporter were, as expected, dependent on caspase-1 and proteasome, respectively (Fig. 3). These properties indicate that the IDOL gene and IDOL mice are useful tools for monitoring the activity of IL-1β. IDOL mice were also shown to be useful for visualization of tissue-specific inflammation (Figs 4 and 5). Therefore, IDOL mice would probably be useful in sensitive detection of local inflammation (the activity of IL-1β in a narrow sense) *in vivo*.

As described in the introduction, several reporter systems that are dependent on the regulation of transcription or processing of IL-1β have previously been developed. However, the reporter we developed is dependent on both regulatory systems and consequently has several advantages. One advantage is the low background signal. In the case where the reporter is regulated solely by the IL-1β promoter, substantial luminescent signals are detected even under noninflammatory conditions. On the other hand, such noise signals are decreased to 1/50 in our reporter, because transcriptional leak-derived reporter proteins should be degraded in the case of dual regulation (Fig. S1). Another advantage of our system is its high responsivity to inflammatory stimulation. We can produce an inflammation responsive reporter by using either transcriptional regulation or processing regulation. By using both regulations, however, we were able to produce a reporter that combines both abilities (Fig. 2e). Such a reporter system should have higher responsivity to inflammatory stimulation than a reported responsive to single regulation.

The activity of the reporter in IDOL mice was constitutively high in the spleen and in the lung (Figs 4 and 5). For this reason, it might be difficult to detect subtle changes of inflammatory conditions and IL-1β activity in those organs, even when using IDOL mice. A comparison of levels of expression of the reporter gene in various tissues showed that it was higher in the spleen and the lung (Fig. S2a). We speculate that this high reporter activity in the spleen and lung is due to high expression levels of the reporter gene in these organs in IDOL mice. As the level of expression of endogenous IL-1β is also high in the spleen and the lung, in common with that of reporter gene (Fig. S2b), the high reporter activity in these organs of IDOL mice accurately reflects the physiological condition *in vivo*.

IDOL mice transduced with a reporter that has a high responsiveness to inflammatory conditions and which accurately reflects physiological conditions might be useful in low-invasiveness monitoring of IL-1β activity *in vivo*. Consequently, we might be able to address various problems associated with inflammation and IL-1β activity in diseases and in pharmaceutical development by using IDOL mice. For example, by mating IDOL mice with some disease mouse model, we could examine temporal change in inflammation and IL-1β activity with the progression of a disease in identical mice. If a certain drug were to be administrated to IDOL mice, we could also easily examine the effects of that drug on inflammation and IL-1β activity at the whole-body level of mice. Recently, many reports indicate that chronic low-grade inflammation is a common pathophysiological condition in various disease, including lifestyle disease (fatty liver, diabetes, obesity, etc), cancer, and autoimmune diseases^22^,^23^. Thus, IDOL mice could be useful in monitoring such chronic inflammations, in drug screening for inflammation-regulating compounds, and in developing a range of treatments for diseases. We therefore expect that IDOL mice will be widely used as a valuable resource in medical science.

## Methods

### Cell culture, transfection, and treatment

RAW264 cells were provided by the RIKEN BRC through the National Bio-Resource Project of the MEXT Japan, and were cultured in accordance with the provider’s instructions. Effectene (Qiagen, Venio, Netherlands) was used to introduce plasmid DNA into the RAW264 cells. To induce inflammatory stimulation, cells were treated with 5 μg/mL LPS for 6 h under serum-free culture condition. LPS was obtained commercially (#L2654; Sigma-Aldrich, Saint Louis, MO).

### Gene constructs

pTKpro-GL4 was made by insertion of TKbasal promoter fragment and GL4 fragment into the SpeI/HindIII sites and HindIII/BamHI sites of pTKX3, respectively. TKbasal promoter fragment was produced by PCR using primer A: 5′-aaa act agt aaa aaa tct aga ggc ccc gcc cag cgt ctt gtc att g-3′, primer B: 5′-aaa aaa aag ctt cgc tgt tga cgc tgt taa gcg-3′, and pTKX3 as a template. GL4 fragment was produced by PCR using primer C: 5′-aaa aag ctt cca cca tgg aag atg cca aaa aca tta ag-3′, primer D: 5′-aaa gga tcc tta tta cac ggc gat ctt gcc gcc ctt ctt g-3′, and pGL4.10 (Promega, Madison, WI) as a template.

pIL1b(−5k)-TKpro-GL4 was made by insertion of mouse IL-1β promoter fragment into the SpeI/XbaI sites of pTKpro-GL4. Mouse IL-1β promoter fragment was produced by PCR using primer E: 5′-aaa act agt tcg tct ttt gag aaa gtc agg gca g-3′, primer F: 5′-aaa act agt cac aag gaa gct tgg ctg gag agg atc-3′, and mouse genomic DNA as a template.

pTKpro-GL4-F-CL1PEST was made by insertion of GL4 fragment (∆stop) and Flag-CL1-PEST fragment into the HindIII/KpnI site and XhoI/NheI sites of pTKpro-GL4. GL4 fragment (∆stop) was produced by PCR using primer C, primer G: 5′-aaa ctc gag aaa aaa ggt acc cac ggc gat ctt gcc gcc ctt c-3′ and pGL4.10 as a template. Flag-CL1-PEST fragment was produced by PCR using primer H: 5′-aaa ctc gag gac tac aag gat gac gat gac aag aat tct gct tgc aag aac tgg ttc-3′, primer I: 5′-aaa gct agc tta gac gtt gat cct ggc gct g-3′, and pGL4.12 (Promega) as a template.

pTKpro-GL4-IL1b(2–269)-F-CL1PEST was made by insertion of the mouse IL-1β (2–269 aa) cDNA fragment into the KpnI/XhoI sites of pTKpro-GL4-F-CL1PEST. The mouse IL-1β (2–269 aa) cDNA fragment was produced by PCR using primer J (5′-aaa ggt acc gca gaa gta cct gag ctc gc-3′), primer K (5′-aaa ctc gag gga aga cac aaa ttg cat ggt g-3′), and mouse total cDNA as a template.

pTKpro-GL4-IL1b(17–216)-F-CL1PEST was made by insertion of the mouse IL-1β (17–216 aa) cDNA fragment into the KpnI/XhoI sites of pTKpro-GL4-F-CL1PEST. The mouse IL-1β (17–216 aa) cDNA fragment was produced by PCR using primer L (5′-aaa ggt acc gat gag aat gac ctg ttc ttt g-3′), primer M (5′-aaa ctc gag aaa ccg ttt ttc cat ctt ctt c-3′), and mouse total cDNA as a template.

pTKpro-GL4-IL1b(67-166)-F-CL1PEST was made by insertion of the mouse IL-1β (67–166 aa) cDNA fragment into the KpnI/XhoI sites of pTKpro-GL4-F-CL1PEST. The mouse IL-1β (67–166 aa) cDNA fragment was produced by PCR using primer N (5′-aaa ggt acc ttg tgg ctg tgg aga agc tgt g-3′), primer O (5′-aaa ctc gag tcc ttg tac aaa gct cat gga g-3′), and mouse total cDNA as a template.

pTKpro-GL4-IL1b(92-141)-F-CL1PEST was made by insertion of the mouse IL-1β (92–141 aa) cDNA fragment into the KpnI/XhoI sites of pTKpro-GL4-F-CL1PEST. The mouse IL-1β (92-141 aa) cDNA fragment was produced by PCR using primer P (5′-aaa ggt acc ttc ttt tcc ttc atc ttt gaa g-3′), primer Q (5′-aaa ctc gag ata tgg gtc cga cag cac gag-3′), and mouse total cDNA as a template.

pIL1b(−5k)-TKpro-GL4-HindIII-IL1b(17–216)-F-CL1PEST was made by insertion of the mouse IL-1β promoter fragment into the SpeI/XbaI sites and the HindIII-mouse IL-1β (17–216 aa) cDNA fragment into the KpnI/XhoI sites of pTKpro-GL4-F-CL1PEST. The mouse IL-1β promoter fragment was produced by PCR using primer E, primer F, and mouse genomic DNA as a template. The HindIII-mouse IL-1β (17–216 aa) cDNA fragment was produced by PCR using primer S (5′-aaa ggt acc aag ctt gat gag aat gac ctg ttc ttt g-3′), primer M, and mouse cDNA as a template.

### Transgenic mice

The 8.9-kb SpeI-SfiI fragment of pIL1b(−5k)-TKpro-GL4-HindIII-IL1b(17-216)-F-CL1PEST was microinjected as a transgene into fertilized mouse eggs (C57BL/6), and the transgenic offspring were screened by PCR using the following primers: 5′-gaa ttc gaa cac gca gat gc-3′ and 5′-tag cgc ttc ata gct tct gc-3′. The experimental protocols that involved animals were approved by the Animal Studies Committees at Kumamoto University and Gunma University (13–024 and 130–030), and all experiments were performed in accordance with the institute guidelines.

### Luciferase reporter assay

RAW264 cells were seeded in 12-well plates at 5 × 10^4^ cells/well, then transfected with plasmid DNA (2 μg/well). 30 h after transfection and 6 h after treatment with LPS, the cells were lysed for luciferase assay. pRL-hTK (Promega) was used as an internal control in this assay. CD11b^+^ cells collected from the spleens of IDOL mice were also seeded in 12-well plates at 5 × 10^4^ cells/well and treated with 5 μg/mL LPS under serum-free culture conditions. 6 h after treatment with LPS, the cells were lysed for luciferase assay. Luciferase activity was measured by using a luciferase assay system (Promega) and a luminometer (Berthold Technologies, Bad Wildbad, Germany).

### RT-PCR and quantitative PCR

Total RNA derived from each tissue of wild-type and IDOL mice were prepared by using Isogen reagent (Nippon Gene, Tokyo, Japan). The cDNA was synthesized by using the Super Script first-strand synthesis system (Invitrogen, Waltham, MA) in accordance with the manufacturer’s instructions. *IDOL* cDNA was amplified by 35 cycles of PCR with 5′-agg act gac cgg caa gtt gg-3′ and 5′-cat gtc ctc atc ctg gaa gg-3′ as primers. *GAPDH* cDNA was also amplified by 35 cycles of PCR with 5′-ctg aac ggg aag ctc act gg-3′ and 5′-cac cac cct gtt gct gta gc-3′ as primers. Quantitative PCR analysis of each transcript was performed by using TaqMan probe and StepOnePlus (Applied Biosystems, Waltham, MA) in accordance with the manufacturer’s instructions. The results of the quantitative PCR analysis are shown as mean ± S.E.M from triplicate experiments for RNA isolated from three independent samples. Probe/primer sets Mm00434228_m1 and 4352339E (Applied Biosystems) were used for quantification of IL-1β and GAPDH transcripts, respectively. IDOL transcripts were quantified by using 5′-act tcg tgc ccg aga gct t-3′ as the forward primer, 5′-ccg gta ctg cca cta ctg ttc a-3′ as the reverse primer, and 5′-FAM-acc ggg aca aaa c-MGB-3′ as the probe.

### Imaging of luminescent signals *in vivo* and *ex vivo*

IDOL mice were injected intraperitoneally with D-luciferin (150 μg/g body weight) in PBS 10 min before imaging analysis. Tissues were collected surgically from the IDOL mice 10 min after luciferin injection and were immersed in 300 μg/mL D-luciferin. CD11b^+^ cells were collected by cell sorting from the spleens of IDOL mice uninjected with D-luciferin and were cultured in a medium containing 300 μg/mL D-luciferin. Imaging analyses were performed by using a cooled CCD camera (IVIS, Perkin Elmer, Waltham, MA). After a grey-scale photograph had been acquired, luminescence images were obtained under the following conditions. For *in vivo* analysis: exposure, 300 s; field of view, 12.5 cm; binning (resolution) factor 4; open filter. For *ex vivo* analysis: exposure, 60 s; field of view, 5 cm, binning (resolution) factor 8; open filter.

### Assays for IL-1β, AST, ALT, and lipase in serum

Blood collected from the tail veins of mice was incubated statically for 10 min at 25 °C to permit clotting. Serum was separated from the blood clot by centrifugation at 10,000 *g* for 10 min at 4 °C. The IL-1β level in the serum was quantified by using commercial ELISA kits (R&D systems, Minneapolis, MN). AST and ALT activities in serum were both measured by using a transaminase assay kit (Wako, Osaka, Japan). Lipase activities in serum were measured by using a lipase assay kit (Wako, Osaka, Japan).

### Histological procedures

Tissues were fixed with 3.7% formaldehyde for 1 day and then washed with PBS. They were then embedded in paraffin and serial sections 4 μm in thickness were cut. The sections were stained with hematoxylin and eosin.

### Collection of CD11b^+^ cells

Spleens were dissected from abdominal cavities of mice and were filtered through a 40-μm nylon strainer. A 0.83% aqueous solution of NH_4_Cl was used to remove red cells. The resulting suspension of single splenic cells was stained with FITC-conjugated anti-CD11b monoclonal antibody (BD pharmingen, San Jose, CA), and the CD11b^+^ cells were collected by using a cell sorter (S3, Bio-Rad, Hercules, CA).

### Western blot analysis

CD11b^+^ cells collected from the spleens of IDOL mice were treated with 20 μM MG132, 5 μg/mL LPS, and/or 20 μM caspase-1 inhibitor for 6 h, or were left untreated. The cells were lysed in SDS sample buffer (50 mM Tris-HCl, pH 6.8, 2% SDS, 50 mM DTT, 10% glycerol, and 1 mg/ml bromophenol blue). The lysate was heated to 98 °C for 10 min, and SDS-PAGE was performed to separate the proteins in the lysate. After electrophoresis, the proteins were electrotransferred onto a poly(vinylidene fluoride) microporous membrane and immunodetected with the appropriate antibody. The following antibodies were used for Western blot analysis: anti-luciferase polyclonal antibody (#G7541; Promega), and anti-FLAG polyclonal antibody (#F7425; Sigma-Aldrich). MG132 (Peptide Institute, Osaka, Japan), LPS (#L2654; Sigma-Aldrich) and caspase-1 inhibitor (#sc-3071; Santa Cruz) were obtained commercially.

## Additional Information

**How to cite this article**: Iwawaki, T. *et al*. Transgenic mouse model for imaging of interleukin-1β-related inflammation *in vivo*. *Sci. Rep*. **5**, 17205; doi: 10.1038/srep17205 (2015).

## Supplementary Material

Supplementary Information
