# Supplementary material for: Transgenic mouse model for imaging of interleukin-1β-related inflammation in vivo
Source: Sci Rep. 2015 Nov 24;5:17205. doi: 10.1038/srep17205 (PMC4657042; doi:10.1038/srep17205)

Supplementary information for

Transgenic mouse model for imaging of interleukin-1β-related inflammation *in vivo*

Takao Iwawaki, Ryoko Akai, Daisuke Oikawa, Takae Toyoshima, Mayuko Yoshino, Mitsumi Suzuki, Naoki Takeda, Tomo-o Ishikawa, Yosky Kataoka, and Ken-ichi Yamamura

Contents

Supplementary Figure 1
Supplementary Figure 2

SUPPLEMENTARY FIGURE LEGENDS

Supplementary Figure 1

Reporter assay to compare the background signal level of an IL-1β promoter-regulated luciferase gene with that of the IDOL gene

Reporter constructs were transfected into RAW264 cells. The transfectants were culture under normal conditions for 30 h before the reporter assay. The results are shown as mean (column) ± S.E.M (error bar) from triplicate experiments.

Supplementary Figure 2

Quantitative PCR analysis to compare the expression levels of the IDOL transgene in various tissues of IDOL mice

(a) Expression level of the IDOL transgene. (b) Expression level of the endogenous IL-1β gene. GAPDH was used as an internal standard. The results are shown as mean (column) ± S.E.M (error bar) from triplicate experiments in each assay.

Supplementary Figure 1

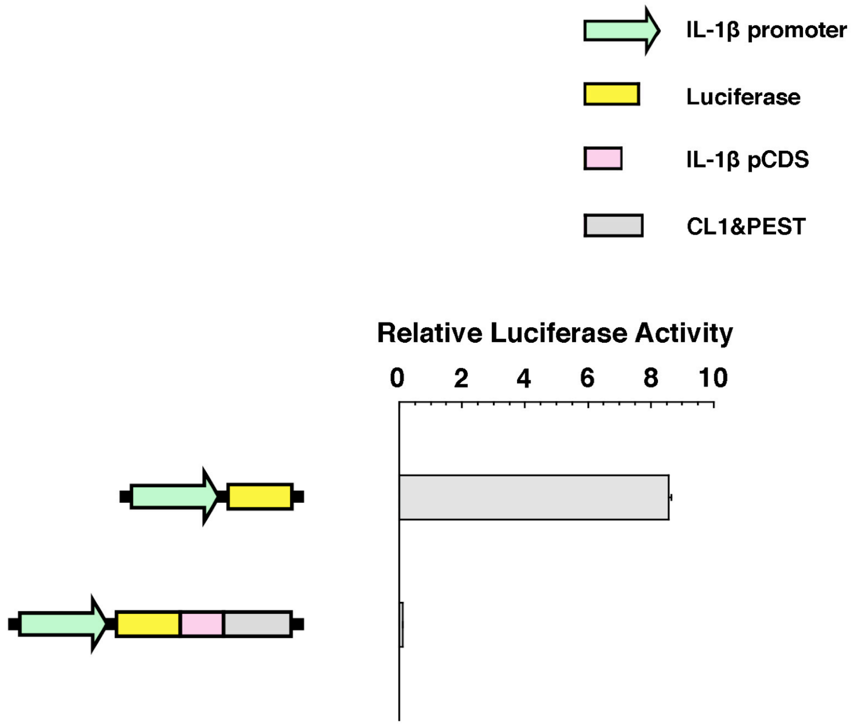

Supplementary Figure 2

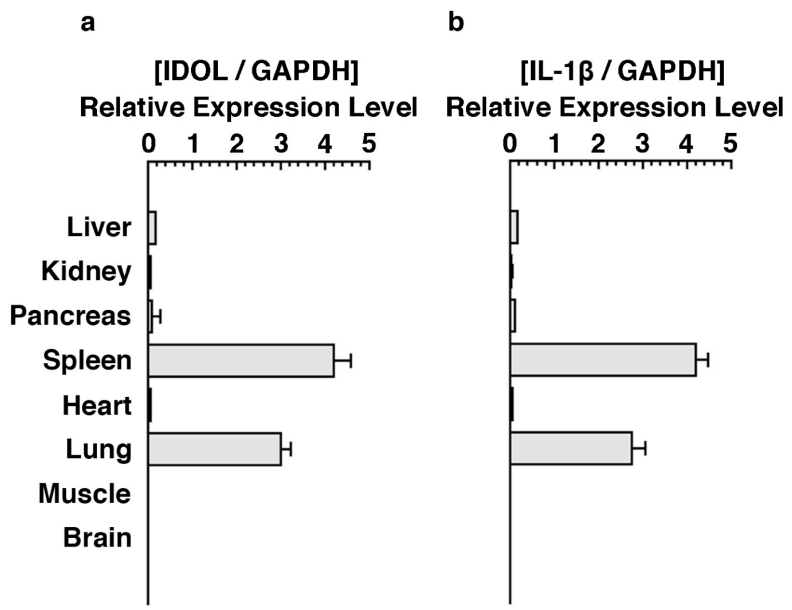

Supplement: Supplementary Information [file srep17205-s1.pdf]
